# An engineered tetra-valent antibody fully activates the Tie2 receptor with comparable potency to its natural ligand angiopoietin-1

**DOI:** 10.1038/s41598-021-93660-4

**Published:** 2021-07-07

**Authors:** Yukari Koya, Hiromi Nara, Shigenori Yagi, Chihoko Ueno, Masazumi Kamohara

**Affiliations:** Drug Discovery Research, Astellas Pharma Inc., Tsukuba, Japan

**Keywords:** Antibody therapy, Permeation and transport, Tight junctions, Vascular diseases

## Abstract

Activation of the tyrosine kinase with Ig and epidermal growth factor homology domain 2 (Tie2) receptor by angiopoietin-1 (Ang1) is critical for vascular stabilization: it promotes survival signal transduction via auto-phosphorylation and reduces vascular permeability by strengthening tight junctions between endothelial cells. Thus, Tie2/Ang1 signaling is a promising therapeutic target for vascular diseases. However, in vivo use of existing Tie2 signaling modulators, such as recombinant Ang1, is restricted by limitations in manufacturability and stability. Here, we present a novel engineered tetra-valent agonistic antibody, ASP4021, which can specifically and fully activate the Tie2 receptor in an equivalent manner to Ang1. ASP4021 induced Tie2 self-phosphorylation and inhibited apoptosis in a human primary endothelial cell line. Additionally, single administration of ASP4021 significantly suppressed mustard-oil-induced vascular permeability in rats. ASP4021 may thus be a potential therapeutic candidate for diseases associated with vascular weakness such as diabetic retinopathy, diabetic macular edema and critical limb ischemia.

## Introduction

Tie2 is mainly expressed in vascular endothelial cells and Ang1, its natural ligand, is a multimer-type-secreted glycoprotein expressed in pericytes. When Ang1 binds Tie2, Tie2 undergoes oligomerization and auto-phosphorylation, which leads to promotion of anti-apoptotic activity in vascular endothelial cells via induction of Akt phosphorylation^1^, inhibition of vascular permeability via suppression of Src signaling^2^, and activation of vascular maturation and vascular remodeling^3,4^. Furthermore, activation of Tie2 in vascular endothelial cells by Ang1 induces vasodilation and enhances blood flow via nitric oxide production^5^. Tie2/Ang1 signaling is thus a promising therapeutic target for diseases associated with vascular weakness such as diabetic retinopathy^6–8^ and critical limb ischemia^9,10^. Despite the promise of some therapeutic candidates for vascular stability, however, clinical use of recombinant Ang1 and its variants, such as the chimeric protein COMP-Ang1, are limited due to difficulties with large scale production, short in vivo half-lives and potential immunogenicity^11–13^.

Agonistic antibodies are an effective alternative to native ligands because technologies for mass production of antibodies are well established. Antibodies are highly stable with long half-lives in circulation and bind specifically to their target protein. The Tie2 agonistic mouse monoclonal antibody 15B8 induces phosphorylation of the Tie2 receptor and promotes survival and angiogenesis of endothelial cells in vitro and in vivo^14^. Furthermore, the Tie2 agonistic fully human antibody 1-4 h facilitates phosphorylation of Tie2 and intracellular signal transmission^15^. While 1-4 h also increases the formation of capillary-like tubes in endothelial cells in vitro, its effects in an in vivo animal model could not be assessed due to a lack of cross-reactivity for rodent Tie2.

Here, we identified agonistic monoclonal antibodies with comparable activity to that of Ang1. Surprisingly, only dimer and higher molecular weight antibodies, but not monomers, showed agonistic activity in Tie2-expressing Ba/F3 cells. We thus hypothesized that tetra-valency might be important for inducing full agonistic activity by facilitating Tie2 receptor dimerization or multimerization for subsequent auto-phosphorylation. As expected, an engineered tetra-valent antibody, but not a general bivalent antibody, showed agonistic and anti-apoptotic activity in Tie2-expressing Ba/F3 cells. Moreover, single subcutaneous administration of the tetra-valent antibody significantly suppressed mustard-oil-induced vascular permeability in rats in vivo. These results proved our hypothesis, indicating that our engineered tetra-valent antibody is an effective substitute for Ang1 in therapeutic applications.

## Results

### Characterization of newly identified anti-Tie2 agonistic antibodies

**Figure 1:**
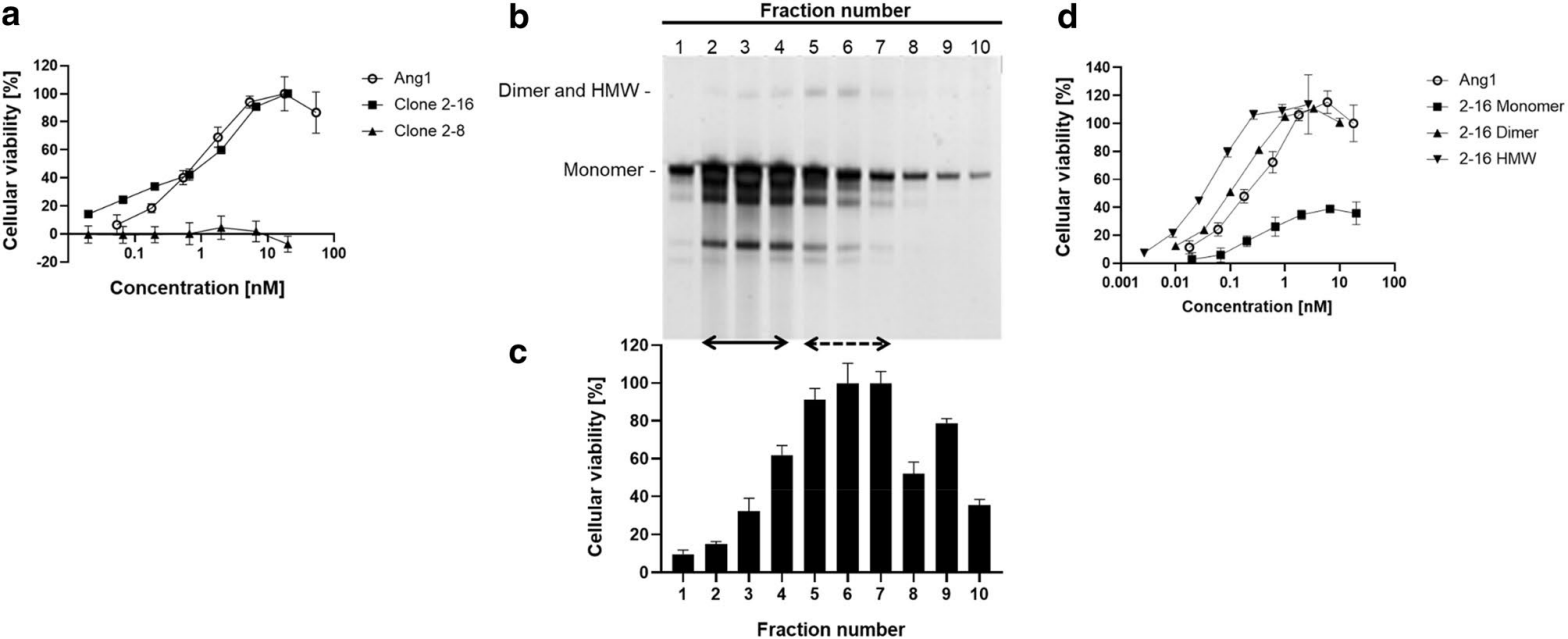
Characterization of newly identified anti-Tie2 agonistic antibodies. (**a**) Cellular viability as an indicator of agonistic activity of anti-Tie2 antibodies produced by hybridoma clones 2–16 and 2–8 were assessed in human Tie2-expressing Ba/F3 compared to Ang1. Cellular viability (%) was determined using a CellTiter-Glo assay, with basal activity without any ligand (PBS) defined as 0%, and the activity of 17.9 nM (1 µg/mL) Ang1 defined as 100%. Data represent mean ± SD. (**b**,**c**) Fractionation of the antibody solution using AEX. (**b**) shows SDS PAGE of each fraction from AEX, and (**c**) shows cellular viability as an indicator of the agonistic activity of each fraction in human Tie2-expressing Ba/F3. Solid (left) arrow indicates antibody-rich fractions, dashed (right) arrow indicates the fractions with higher agonistic activity. Cellular viability (%) was determined using a CellTiter-Glo assay, with basal activity without any ligand (PBS) defined as 0%, and the activity in fraction 6 defined as 100%. (**d**) Cellular viability as an indicator of the agonistic activity of purified 2–16 monomer, dimer and HMW human monoclonal antibodies, isolated using SEC (Supplementary Fig. S1 and S2), in human Tie2-expressing Ba/F3 compared to Ang1. Cellular viability (%) was determined as described in (**a**).

First, we immunized VelocImmune mice with a recombinant human Tie2-Fc chimeric protein. Using a standard method, lymphocytes from immunized mice were fused with mouse-derived myeloma cells to generate hybridomas that produce anti-Tie2 antibodies. To select antibodies that bind to the natural conformation of the Tie2 receptor, cell-based ELISA was performed using Tie2-expressing CHO cells. Monoclonal antibodies bind to human Tie2 with cross-reactivity for monkey, rat, and mouse Tie2 were selected using cell-based ELISA with CHO cells expressing Tie2 from each species. Second, to screen for antibodies with natural ligand-like properties, a competitive binding assay was conducted using recombinant human Tie2-Fc with labeled COMP-Ang1. Antibodies that compete against COMP-Ang1 likely bind to a similar binding site to Ang1 on Tie2. Additionally, because Ang1 and Ang2, a natural antagonist of Tie2, have the same affinity for the same binding site on Tie2^16^, selected antibodies should also show competitive activity against Ang2. Third, to identify a functional agonistic antibody, cellular viability as an indicator of agonistic activity was evaluated using the human Tie2-expressing mouse pro-B cell line Ba/F3. While original Ba/F3 depends on exogenous interleukin-3 (IL-3) for survival, this IL-3 dependency can be compensated by ectopic overexpression of a ligand-stimulated or constitutively active tyrosine kinase^17^. The survival of Tie2-expressing Ba/F3, evaluated here using a CellTiter-Glo assay, depends on Tie2 agonistic stimulation in the absence of IL-3. While the monoclonal antibody clone 2–16 showed comparable cellular viability to recombinant Ang1, monoclonal antibody clone 2–8 did not (Fig. 1a). Interestingly, some purified antibodies showed unstable and transient agonistic activity (cellular viability) that disappeared after a couple of weeks. To examine these intriguing findings, anion-exchange column chromatography (AEX) was first performed to eliminate the possibility that agonistic activity may have been derived from contamination. Surprisingly, antibody-rich fractions (fraction number 2 to 4) showed weak cellular viability, whereas fractions with higher viability (fraction number 5 to 7) contained smaller amounts of antibodies and unknown high molecular weight (HMW) proteins (Fig. 1b,c). We hypothesized that the unknown HMW proteins were aggregated antibodies and would have higher viability. It is known that some antibodies aggregate during purification using a Protein-A/G column^18^. To investigate our hypothesis, we performed size exclusion chromatography (SEC) analysis. We confirmed that the purified antibody solution contained monomers, dimers and HMW antibodies, and separated this solution to obtain three fractions containing monomers, dimers or HMW proteins with high purity (Supplementary Fig. S1 and S2). Next, we evaluated cellular viability as an indicator of agonistic activity in three fractions containing monomers, dimers and HMW proteins from antibody clone 2–16 (Fig. 1d). While the dimer and HMW fractions showed full agonistic activity equivalent to native Ang1, the monomer fraction only showed partial activity. This result confirms our hypothesis and suggests that antibodies with four or higher valence acquired agonistic activity through Tie2 multimerization.

### Generation and in vitro evaluation of the tetra-valent anti-human Tie2 antibody

**Figure 2:**
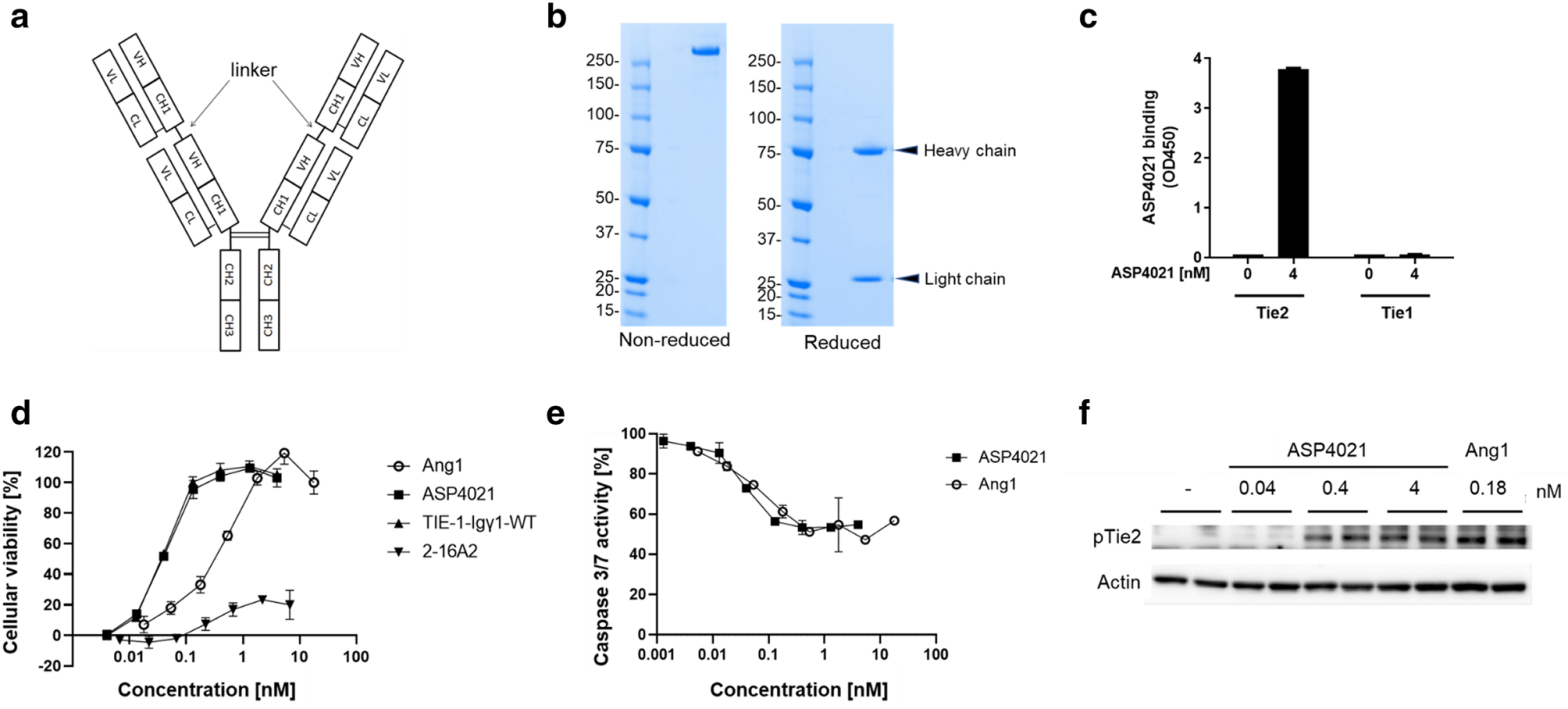
Generation and in vitro evaluation of the tetra-valent anti-human Tie2 antibody. (**a**) Structure of the tetra-valent antibody. (**b**) SDS-PAGE of the tetra-valent antibody under non-reduced (left) and reduced (right) conditions. Molecular weights (kDa) are indicated on the left of each gel. (**c**) Binding activity of ASP4021 to human Tie2 and Tie1 by direct ELISA. ASP4021 binding is shown as the absorbance at OD450. Each value is expressed as mean ± SEM. (**d**) Cellular viability as an indicator of agonistic activity of the tetra-valent and bi-valent antibodies in human Tie2-expressing Ba/F3 compared to Ang1. Cellular viability (%) was determined as described Fig. 1a. (**e**) Anti-apoptotic activity of ASP4021 in human Tie2-expressing Ba/F3 compared to Ang1. Caspase 3/7 activity (%) was calculated using the Caspase-Glo 3/7 assay, with basal activity with IL-3 defined as 0%, and activity without any ligand (PBS) defined as 100%. Data represent mean ± SD. (**f**) Phosphorylation of Tie2 induced by ASP4021 in HUVEC compared to Ang1. HUVEC were treated with ASP4021 or recombinant Ang1 for 30 min. Levels of phosphorylated Tie2 and actin in cell lysates were detected using Western blotting. The assay was performed in duplicate. Full-size versions of Western blots are depicted in Supplementary Fig. S3.

We hypothesized that this tetra-valency might be important for full agonistic activity by facilitating Tie2 receptor dimerization or multimerization for subsequent auto-phosphorylation. To produce antibodies with full agonistic activity, anti-Tie2 antibody clones were genetically engineered to form tetra-valent human antibodies. The tetra-valent antibody consisted of two heavy chains and four light chains (Fig. 2a). Each heavy chain comprised two structures, one with a heavy chain variable region (VH) followed by a CH1 region and the other with VH, CH1, CH2 and CH3 regions of the human IgG1 heavy chain gene, joined by a linker. Each light chain comprised a variable region (VL) and constant region (CL) of the human immunoglobulin kappa light chain gene. For linker optimization, the IgG1 upper hinge sequence-based linker EPKSCGS, IgG3 upper hinge sequence-based linker ELKTPLGDTTHTGS and IgG3 upper hinge sequence-based long linker ELKTPLGDTTHT(GGGGS) × 10 were prepared. Because all three showed equivalent agonistic efficacy, EPKSCGS was adopted. The engineered tetra-valent human antibody generated from clone 2–16 was called TIE-1-Igγ1-WT, and the bivalent human antibody was called 2-16A2. Amino acid mutations of L234A, L235A and P331S were introduced into the heavy chain constant region to reduce antibody-dependent cellular cytotoxicity or complement-dependent cytotoxicity^19–22^ because the target molecule, Tie2, is a cell surface antigen. The engineered tetra-valent antibody generated from clone 2–16 with mutations in the heavy chain was called ASP4021. The tetra-valent antibodies could be stably produced using the 293 or CHO cell systems, and purified using affinity chromatography and SEC. SDS PAGE indicated that the tetra-valent antibody had the expected molecular weight (Fig. 2b) and structure (Fig. 2a). The binding activity of ASP4021 for recombinant Tie2-Fc protein from four animal species (human, mouse, rat and monkey) was analyzed using a Biacore system based on a surface plasmon resonance technique (Supplementary Table). The dissociation constant (K_D_) for the interaction between ASP4021 and human, mouse, rat and monkey Tie2 was 1.5 × 10^–9^, 1.0 × 10^–9^, 1.3 × 10^–9^ and 1.5 × 10^–9^ mol/L, respectively. ASP4021 showed equivalent and substantially high binding activity to Tie2 from all four species. We checked the binding specificity by comparing with binding to Tie1, another Ang1 receptor. Direct ELISA showed that ASP4021 did not bind to recombinant human Tie1 (Fig. 2c). While both tetra-valent TIE-1-Igγ1-WT and ASP4021 showed similarly potent cellular viability to Ang1 in human Tie2-expressing Ba/F3, bivalent 2-16A2 (purified as a monomer) showed weak activity (Fig. 2d). This result proved our hypothesis that genetically engineered tetra-valent antibodies, TIE-1-Igγ1-WT and ASP4021, can induce similarly potent agonistic activity to the natural ligand Ang1.

To evaluate whether ASP4021 induces anti-apoptotic activity, the Caspase-Glo 3/7 assay was used to evaluate caspase 3/7 activity in Tie2-expressing Ba/F3. ASP4021 showed comparable anti-apoptotic activity to Ang1 (Fig. 2e). To validate the agonistic activity in human primary cells, Tie2 phosphorylation was evaluated in human umbilical vein endothelial cells (HUVEC). Levels of pTie2 after treatment with ASP4021 at 100 ng/mL (0.4 nM) increased 7.1-fold compared to vehicle control (Fig. 2f and Supplementary Fig. S3). Ang1 induced a maximum 7.0-fold increase in pTie2 at 0.18 nM (10 ng/mL). Thus, ASP4021 induces anti-apoptotic activity through phosphorylation of Tie2 in Ba/F3 cells and human primary cells in the same manner as Ang1. In addition, we have already obtained data showing that ASP4021 significantly attenuates IL-1b-induced permeability in HUVEC barrier function (Supplementary Fig. S4). These results indicates the effectiveness of the antibody during injury in a human cell model.

### Effect of ASP4021 and related antibodies on vascular permeability in vivo

**Figure 3:**
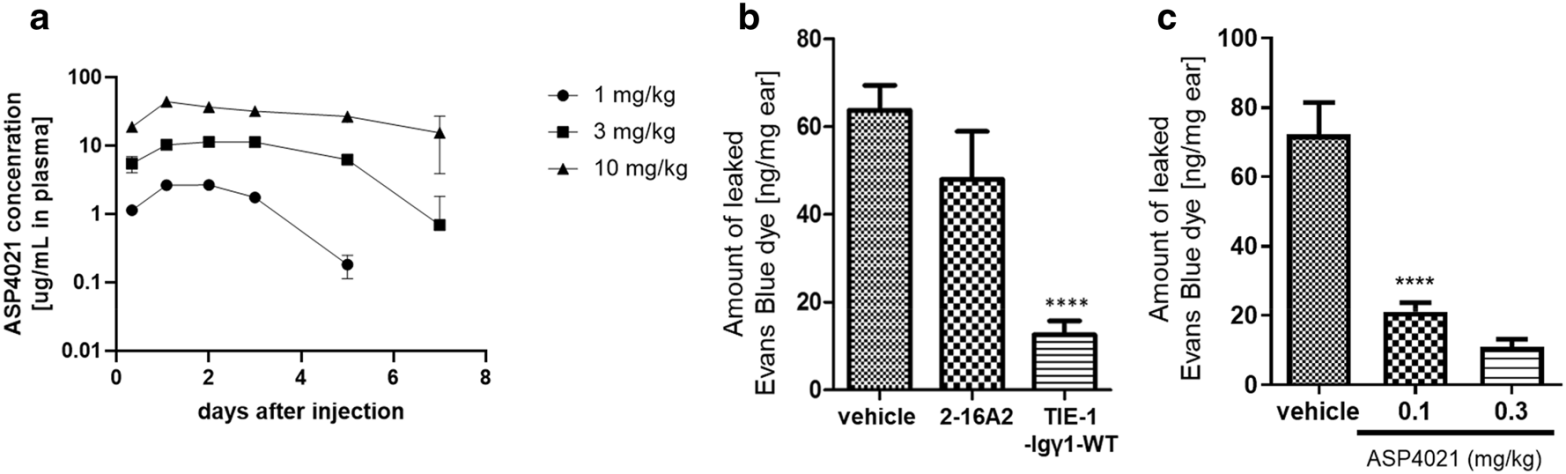
Effect of ASP4021 and related antibodies on vascular permeability in vivo. (**a**) Pharmacokinetics analysis of ASP4021 in rats. The concentration of ASP4021 in rat plasma after subcutaneous administration was measured using an ELISA-based assay. Each value indicates the mean ± SD of 3 rats per group. (**b**) Inhibitory effect of 2-16A2 and TIE-1-Igγ1-WT on vascular permeability in a rat mustard oil-induced vascular permeability model following treatment with 0.3 mg/kg. The vertical axis indicates the amount of leaked Evans Blue dye. The graph indicates the mean ± SEM of 6 rats per group. Student’s t-test was employed for statistical analysis. ****p < 0.0001 vs the vehicle group. (**c**) Inhibitory effect of ASP4021 on vascular permeability in a rat mustard oil-induced vascular permeability model following treatment with 0.1 or 0.3 mg/kg. Each graph indicates the mean ± SEM of 6 rats per group. Dunnett’s multiple comparisons test was employed for statistical analysis. ****p < 0.0001 vs the vehicle group.

Before initiating an in vivo study, the pharmacokinetic analysis was conducted to measure the plasma concentration after subcutaneous administration to rats (Fig. 3a). The result showed that ASP4021 was stable in blood for more than one week. When the blood concentration fell below around 1 µg/mL, ASP4021 was rapidly eliminated from plasma and showed non-linear pharmacokinetics. We predict that the non-linear pharmacokinetics may be explained by target-mediated drug disposition, which is common for monoclonal antibody drugs, especially those targeting membrane receptors^23^.

Finally, we evaluated the antibodies in an in vivo mustard oil-induced vascular permeability rat model, which is the modified Miles assay^24^. In a previous study, Ang1 showed inhibitory activity against vascular hyper-permeability^25^. First, we compared tetra-valent TIE-1-Igγ1-WT and bivalent 2-16A2 with vehicle control. The antibodies were subcutaneously administered at 0.3 mg/kg 48 h before mustard oil or mineral oil application to the rats’ ears. Evans Blue dye was systemically administered just before oil application to measure vascular permeability. The amount of leaked Evans Blue dye extracted from ears 30 min after oil application was measured. As shown in Fig. 3b, compared to the vehicle group, the tetra-valent antibody TIE-1-Igγ1-WT significantly inhibited dye leakage, while bivalent 2-16A2 did not. The blood concentration of 2-16A2 and TIE-1-Igγ1-WT 48 h after administration, the time point at which vascular permeability was evaluated, was 1.3 ± 0.05 nM and 0.03 ± 0.003 nM, respectively. Based on estimations from the viability assay for predicting agonistic activity using Tie2-expressing Ba/F3 (Fig. 2d), the concentration of each antibody was sufficient to exert agonistic activity.

Evaluation of ASP4021 in this model showed that ASP4021 dose-dependently inhibited vascular hyper-permeability (Fig. 3c). It was not possible for us to simultaneously compare the efficacy of ASP4021 and Ang1 due to the short half-life of Ang1 in vivo. ASP4021 showed approximately 80% inhibition of vascular hyper-permeability compared to vehicle, which is higher than that shown by an Ang1-expressing adenovirus (50% inhibition) in this model^25^.

## Discussion

Our results demonstrate that dimer and higher multimeric antibodies, but not monomer antibodies, show agonistic activity to Tie2. Interaction of Tie2 with highly oligomeric Ang1 leads to receptor oligomerization and activation^26^. Although it is not fully understood how ligand binding-induced oligomerization of Tie2 leads to receptor activation, Ang1-induced Tie2 oligomerization is thought to be critical for activating Tie2 via auto-phosphorylation and initiating downstream signaling. However, purified dimer and higher multimeric antibody fractions are generally unstable, and are therefore unsuitable for therapeutic use in vivo and mass production. We generated tetra-valent anti-Tie2 antibodies by fusing two Fab regions head-to-tail^27,28^. Lutikizumab (ABT-981), a dual variable domain that simultaneously binds IL-1α and IL-1β, was developed using the same format. In a clinical study, lutikizumab was well tolerated and behaved similarly to conventional monoclonal antibodies with a half-life of 10 to 14 days^27^. We have confirmed that ASP4021 has good pharmacokinetic properties in rats and non-human primates (data not shown), and expect it to also be well tolerated and stable in humans. ASP4021 was efficiently produced using both CHO and 293 cell systems, which are commonly used for mass production of therapeutic antibodies. Thus, ASP4021 overcomes the manufacturability and stability limitations of Ang1.

In previous studies, anti-Tie2 antibodies, 15B8 and 1-4 h, showed agonistic activity in the bi-valent antibody form. However, whereas ASP4021 showed cross-reactivity for rodent Tie2, neither 15B8 nor 1-4 h bound to rodent Tie2^15^, suggesting that the epitope recognized by ASP4021 on the surface of Tie2 differs from that of the previous antibodies. The epitope recognized by an antibody might be important for its agonistic activity, given that not all antibody clones in our study showed agonistic activity even when genetically engineered into tetra-valent antibodies. We are currently performing detailed analysis of the epitope of ASP4021. We expect that the epitope of ASP4021 will be similar to the binding site of Ang1 on Tie2, because its clonal prototype 2–16 competed with labeled Ang1 in the antibody screening step. Further, like Ang1, Ang2 also interacts exclusively with the Ig2 domain of Tie2 in a similar manner^29,30^. Therefore, ASP4021 likely competes against Ang2, a natural antagonist of Tie2 that is involved in vascular leakage and abnormal vessel structure and is elevated in the plasma of diabetic retinopathy patients^8^, making it a potential therapeutic target for retinal disease. Several drug candidates targeting Ang2 are under investigation in clinical trials. Faricimab, a bispecific antibody that inhibits VEGF-A and Ang2, is in phase 3 trials for neovascular age-related macular degeneration and diabetic macular edema^31^. Nesvacumab, a monoclonal antibody that inhibits Ang2, was examined in combination with aflibercept to determine the potential for additional benefits over aflibercept monotherapy in phase 2 studies; however, the trial failed^31^. Because the plasma concentration of Ang2 rises under ischemic disease conditions, it may be difficult for Ang2-targeting drugs to stably activate the Tie-2/angiopoietin pathway. ASP4021 can directly modulate the Tie2/angiopoietin pathway, and its effect is unlikely to be affected by the concentration of Ang2. We think our approach is the best way to treat ischemic diseases.

To investigate the efficacy of ASP4021 in these diseases, we are conducting evaluations in a pericyte loss model as a disease model of diabetic retinopathy and diabetic macular edema. In addition, we have already obtained data showing that ASP4021 significantly attenuates IL-1b-induced permeability in HUVEC barrier function (Supplementary Fig. S4). These results are expected to pave the way for new treatment methods for diseases associated with vascular weakness such as diabetic retinopathy, diabetic macular edema and critical limb ischemia.

## Methods

### Ethics statement

All methods were carried out in accordance with the institutional guidelines approved by Astellas Pharma as well as relevant guidelines and regulations. All animal experiments were approved by the Institutional Animal Care and Use Committee (IACUC) of Astellas Pharma and performed in accordance with the guidelines of the Care and Use of Laboratory Animals at Astellas Pharma as well as in full compliance of the ARRIVE guidelines. Tsukuba Research Center was awarded Accreditation Status by the AAALAC International.

### Animals

SD rats (male, 4–5-weeks-old) were obtained from Charles River Laboratories (Japan).

### Cell culture

The human Tie2-expressing mouse pro-B cell line Ba/F3 was established by Dr. Nobuyuki Takakura^32^ and cultured in RPMI1640 supplemented 10% fetal bovine serum (FBS), 1% penicillin–streptomycin and 1% mouse IL-3 culture supplement. Another human Tie2-expressing Ba/F3 cell line was established at Astellas Pharma by transfecting a human Tie2-expression plasmid into Ba/F3 (RIKEN; RCB0805), selecting using blasticidin, and culturing in RPMI1640 supplemented with 10% FBS, 1% penicillin–streptomycin, 1% mouse IL-3 culture supplement and 12 μg/mL blasticidin. Human umbilical vein endothelial cells (HUVEC) were purchased from Lonza (C2517AS; Lot. No. 8F3288) and cultured in EGM-2 basal medium containing growth factors (Lonza; CC-3162).

### Immunization and screening of anti-Tie2 antibodies

Monoclonal antibodies against Tie2 were produced by immunizing VelocImmune mice (Regeneron), which are genetically modified to produce human antibodies. For immunization, a recombinant human Tie2-Fc chimeric protein (R&D Systems) was injected into the VelocImmune mice together with an adjuvant for causing an immune reaction. Using a standard method, the lymph nodes of the immunized mice were extracted, and the lymphocytes were collected and cell-fused with mouse-derived myeloma cell SP2/0 (ATCC; CRL-1581), to prepare a hybridoma. The hybridoma was cloned by limiting dilution and each clone was cultured in CD Hybridoma Medium (Thermo Fisher Scientific), a serum-free culture medium. The antibodies were purified from the culture supernatant using a Protein G column or HiTrap MabSelect SuRe column (GE Healthcare). Anion exchange column chromatography was performed using a Resource Q column (GE Healthcare) with a linear gradient of 0–0.5 mol/L NaCl in 50 mmol/L Tris–HCl, pH 8.0 at a flow rate of 1.0 mL/min, and 0.5 mL/tube fractions were collected. The binding of each antibody to human, monkey, rat, and mouse Tie2 was evaluated by cell ELISA assay using CHO cells expressing each Tie2 protein. Competitive binding activity of the test antibody to Tie2 was evaluated against COMP-Ang1 using competitive ELISA. Briefly, an expression vector containing COMP-Ang1 was introduced into HEK293 cells. COMP-Ang1 was purified from the culture supernatant and biotin-labeled and mixed with the purified antibody, and the mixture was added to a plate immobilized with recombinant human Tie2-Fc chimeric protein. Streptavidin-labeled HRP followed by TMB color developing reagent were added to detect biotin-labeled COMP-Ang1. To stop the reaction, 2 mol/L sulfuric acid was added. Subsequently, absorbance at 450 nm was measured to evaluate competitive binding of the test antibody against COMP-Ang1.

### Cellular viability assay to determine agonistic activity for Tie2 signaling

Human Tie2-expressing Ba/F3 cells were suspended in RPMI1640 medium (Thermo Fisher Scientific) supplemented with 0.05% FBS at 2 × 10^5^ cells/mL and 80 μL was added to each well of a 96-well plate (Sumitomo Bakelite). Thereafter, 20 μL of the purified antibody or Ang 1 diluted with phosphate buffer saline (PBS) was added. After culturing for 72 h in a CO_2_ incubator set to 37 °C, 50 μL of the cell suspension was transferred to a white 96-well plate (Thermo Fisher Scientific). To measure intracellular ATP using CellTiter-Glo Luminescent Cell Viability Kit (Promega), 50 μL of CellTiter-Glo reagent was added into each well. The microplate was shaken and incubated in the dark. Luminescence was measured using EnVisionHTS Multilabel Reader (PerkinElmer).

### Generation of a genetically engineered fully human tetra-valent antibody

The genes encoding the heavy and light chains of the antibody were cloned from a hybridoma. After sequencing the antibody, the framework region of the light and heavy chains was replaced to improve the antibody’s physicochemical properties and stability. To construct an expression vector containing a fully human anti-Tie2 antibody, a gene encoding a signal sequence^33^ and a gene encoding the constant region of human Igγ1 were linked to the 5’ and 3’ ends, respectively, of the gene encoding the heavy chain variable region, and the heavy chain gene was inserted into a GS vector pEE6.4 (Lonza). Further, a gene encoding a signal sequence and a gene encoding the constant region of the human kappa chain were connected to the 5’ and 3’ ends, respectively, of the gene encoding the light chain variable region. This light chain gene was inserted into a GS vector pEE12.4 (Lonza). To construct an expression vector containing a tetra-valent antibody, a structure consisting of a heavy chain variable region and a CH1 region was linked to the N terminus of the fully human anti-Tie2 antibody heavy chain with amino acid mutations L234A, L235A and P331S through a linker and inserted into pEE6.4 with a signal sequence. For the light chain, the expression vector for the fully human antibody described above was used. The GS vectors into which the genes of the heavy and light chains of the antibody had been each inserted were digested with restriction enzymes NotI and PvuI, and ligated using a Ligation-Convenience Kit (Nippon Gene) or the ligation reagent, Ligation high Ver. 2 (TOYOBO). To express the antibodies, expression vectors were transfected into FreeStyle 293 cells (Thermo Fisher Scientific), Expi 293 cells (Thermo Fisher Scientific) or CHO-K1SV cells (Lonza) and cultured for 5–7 days. Antibodies were purified from each of the culture supernatants using a Protein A column, a Protein G column or MabSelect SuRe (GE Healthcare) followed by size exclusion chromatography as described above. Consequently, the ASP4021 solution comprised 99.26% monomers.

### Biacore analysis

Recombinant human, rat and mouse Tie2-Fc chimeric protein (R&D Systems) and recombinant monkey Tie2-Fc chimeric protein (Sino Biological) were each immobilized on a sensor chip CM5 (GE Healthcare) using an Amine Coupling Kit (GE Healthcare) and HBS-EP + buffer (GE Healthcare) in accordance with the manufacturer’s instructions. On the blank cell (flow cell 1), injection of the antigen solution was omitted. Measurement was performed three times each with four types of antigens using Biacore T200 (GE Healthcare). Kinetics data were obtained by injecting increasing concentrations of antibody into the sensor chip at a flow rate of 50 µl/min. The measurements were carried out in HBS-EP + buffer. Contact time and dissociation time were 120 s and 300 s, respectively. Data analysis was performed using Biacore T200 evaluation software version 3.0 (GE Healthcare) by fitting the results to sensorgrams obtained from a bivalent binding model.

### Direct ELISA

A recombinant human Tie1-Fc chimeric protein or human Tie2-Fc chimeric protein (R&D Systems) was prepared in PBS, added to a microplate and incubated at 4 °C overnight. The immobilized solution was removed, and 20% Blocking One (Nacalai Tesque) containing Tris-buffered saline with 0.05% Tween (TBS-T) was added and left to stand at room temperature for 1 h. ASP4021 diluted with 5% Blocking One containing TBS-T was added and incubated at room temperature for 1.5 h, and then washed with TBS-T. A goat anti-human kappa-HRP (Southern Biotech) secondary antibody was added and incubated at room temperature for 1 h, before washing with TBS-T. TMB + One-Step Substrate System (Agilent) was then added to each well. The solution turned blue after incubating for about 20 to 30 min. To stop the reaction, 1 mol/L sulfuric acid was added to each well, turning the solution yellow. Absorbance at 450 nm was measured using Infinite M200 Pro (Tecan).

### Anti-apoptotic assay

Human Tie2-expressing Ba/F3 cells established at Astellas Pharma were washed three times with medium and suspended in RPMI1640 with GlutaMax and 10% FBS. A total of 1.6 × 10^4^ cells/80 μL was added to a 96-well Multi-well Plate for Suspension Culture (Sumitomo Bakelite). ASP4021 or Ang1 (R&D Systems) was serially diluted with PBS, and 20 μL of ASP4021 solution, Ang1 solution or PBS alone was added to each well of the culture plate. The culture plate was incubated for 2 days in a 5% CO_2_ incubator at 37 °C and then at room temperature for 10 min. The cells were suspended by pipetting and transferred (50 μL) to a 96-well white microplate (Thermo Fisher Scientific). To measure caspase-3 and -7 activity for evaluation of apoptosis, 50 μL of fourfold diluted Caspase-Glo3/7 assay solution (Promega) with Tris-buffered saline and 10 mmol/L DTT (Wako), 10 mmol/L CDTA (Nacalai Tesque), 50% glycerol (Nacalai Tesque) and 5% TritonX-100 (MP Biomedicals) were added to each well. The microplate was shaken and incubated for 30 min in the dark. Luminogenic caspase-3/7 substrate, which is proportional to the amount of caspase activity, was measured using an EnVision HTS Multilabel Reader (PerkinElmer).

### Phosphorylation of Tie2 in human umbilical vein endothelial cells (HUVEC)

HUVEC were seeded in a 10 cm dish at 1.5 × 10^6^ cells in EGM-2 basal medium containing growth factors. Cells were washed once with PBS and serum-starved in EGM-2 medium for 2 h before being exposed to 0.18 nM (10 ng/mL) of Ang1 or a final concentration of 0.04, 0.4, or 4 nM (10, 100, 1000 ng/mL) of ASP4021 for 30 min. PBS was used as a vehicle control. Following the treatment, cells were washed once with cold PBS and lysed with RIPA buffer (Sigma), 1 × Halt protease and phosphatase inhibitor (Thermo Fisher Scientific), and 50 U/mL benzonase nuclease (Merck Millipore). After 30 min on ice, lysates were centrifuged, and the supernatant was collected. Protein concentrations in the supernatant were determined using a BCA protein assay kit (Thermo Fisher Scientific). The samples (8.5 μg protein/lane) were reduced and separated by electrophoresis, before being transferred onto an Invitrolon PVDF sheet (Thermo Fisher Scientific). After blocking with Blocking One (Nacalai Tesque), each membrane was incubated with antibodies against pTie2 (Abcam; ab151704) or actin (Abcam; AB8227) at 4 °C overnight. After washing, membranes were incubated with an anti-rabbit (GE Healthcare) and each protein was detected using the chemiluminescence reagent Clarity Western ECL substrate (Bio-Rad) with a CCD camera (Bio-Rad; ChemiDoc). Signal intensity was quantified using Image Labs Software v5.2 (Bio-Rad). The assay was performed once in duplicate. Mean values of pTie2 in each concentration group relative to those of the vehicle control group were calculated from the normalized pTie2 values of each sample.

### Pharmacokinetic assay

ASP4021 was subcutaneously administered to SD rats. Blood was collected from each rat at 8 and 26 h, and 2, 3, 5 and 7 days after administration of the antibody, and plasma was separated. The concentration of ASP4021 in plasma was measured using human Tie2-Fc direct ELISA.

### Mustard oil-induced vascular permeability model

TIE-1-Igγ1-WT, 2-16A2 or ASP4021 diluted with PBS was subcutaneously administered to SD rats. At 48 h after administration of the antibody, Evans Blue dye dissolved in physiological saline (Sigma) was intravenously administered. Immediately, 20 μl of allyl isothiocyanate (also known as mustard oil; Nacalai Tesque) diluted with 5% mineral oil (Sigma) was applied to one ear, and mineral oil to the contralateral ear. After 30 min, both ears were sampled, weighed, then immersed in 1 mL of formamide, and incubated at 70 °C overnight to extract Evans Blue dye from the ear tissue. The Evans Blue dye concentration was determined from the absorbance (measurement wavelength of 620 nm and control wavelength of 740 nm) of the extract. The amount of leakage per ear weight was calculated by dividing the Evans Blue dye concentration by the weight of the ear. The final amount of leaked Evans Blue dye from each animal was calculated by subtracting the amount of leaked Evans Blue dye from the ear that received mineral oil from the amount from the ear that received mustard oil in the same animal. The amount of leaked Evans Blue dye was used as an index of vascular permeability.

### Statistical analyses

Data are presented as mean ± SD or mean ± SEM. In the mustard oil-induced vascular permeability model, significant differences between the test and vehicle groups were determined using Student’s t-test or Dunnett’s multiple comparisons test in GraphPad Prism version 8.0.2. *P* < 0.05 was considered statistically significant.

## Supplementary Information

Supplementary Information.
