# Supplementary material for: An engineered tetra-valent antibody fully activates the Tie2 receptor with comparable potency to its natural ligand angiopoietin-1
Source: Sci Rep. 2021 Jul 7;11:14021. doi: 10.1038/s41598-021-93660-4 (PMC8263585; doi:10.1038/s41598-021-93660-4)
Supplement: Supplementary file 1 — Supplementary Information. [file 41598_2021_93660_MOESM1_ESM.docx]

Supplementary Information

**An engineered tetra-valent antibody fully activates the Tie2 receptor with comparable potency to its natural ligand angiopoietin-1**

Yukari Koya, Hiromi Nara, Shigenori Yagi, Chihoko Ueno, and Masazumi Kamohara*

**Table of Contents**

**1. Supplementary Methods**

1-1. Size exclusion chromatography (SEC) analysis and purification of each fraction

1-2. IL-1-induced permeability assay in HUVEC

**2. Supplementary Results**

**Supplementary Figure S1** Fractionation of antibodies using size exclusion chromatography (SEC)

**Supplementary Figure S2** Purity analysis of monomer, dimer and high molecular weight (HMW) antibodies using SDS-PAGE and SEC

**Supplementary Figure S3** Full-size images of Western blots of phosphorylated Tie2 and actin shown in Fig. 2f

**Supplementary Figure S4** Inhibition of IL-1β-induced permeability by ASP4021 in HUVEC

**Supplementary Table** Binding activity of ASP4021 to human, mouse, rat, and monkey Tie2

**1. Supplementary Methods**

# 1-1. Size exclusion chromatography (SEC) analysis and purification of each fraction

For antibody purification and fractionation by SEC, the affinity chromatography-purified antibody was injected into a HiLoad 26/60 Superdex 200 PG column (GE Healthcare) equilibrated and eluted with PBS at a flow rate of 2.5 mL/min using AKTA explorer 100 and Unicorn 5.11 (Build 407) software (GE Healthcare). Each fraction was collected at 5 mL/fraction into tubes. Subsequently, HMW and dimer fractions were concentrated using Amicon Ultra 10K (Millipore), each fraction was filtered through a SteriFlip 0.22 μm PVDF membrane (Millipore) and the protein concentration was determined using a NanoDrop (Thermo Fisher Scientific). For purity analysis by SEC, 50 µg of antibody was injected into a TSK gel G3000SW (TOSOH) equilibrated and eluted with PBS at a flow rate of 0.5 mL/min using a Waters HPLC system with a separation module (Model 2695), a PDA detector (Model 2996), and Empower 3 data acquisition system.

1-2. IL-1β-induced permeability assay in HUVEC

Permeability was assessed using the CultreCoat 96-well *in vitro* vascular permeability assay kit (3475-096-K; Travigen). Fresh medium was added to the top and bottom chambers and maintained at 37°C in a humidified atmosphere containing 5% CO_2_. HUVEC were seeded at 1 × 10^5^ cells/well in the top chamber and incubated for 48 hours. After changing the medium to starvation medium (containing 0.5% FBS), Ang1, 2-16A2 or ASP4021 or Ang1 were added to the cells at a final concentration of 1 μg/mL in the top and bottom chambers and incubated for 30 minutes. Human IL-1β (R&D Systems) was then added at a final concentration of 20 ng/mL to the top and bottom chambers and incubated for 20 hours. Fluorescein isothiocyanate (FITC)-dextran (Travigen) was added to the treated cells in the top chamber for 30 minutes. The medium in the bottom chamber was transferred to a 96-well plate and fluorescence (485 nm excitation, 520 nm emission) was measured using the Safire2 plate reader (Tecan).

**2. Supplementary Results**

**
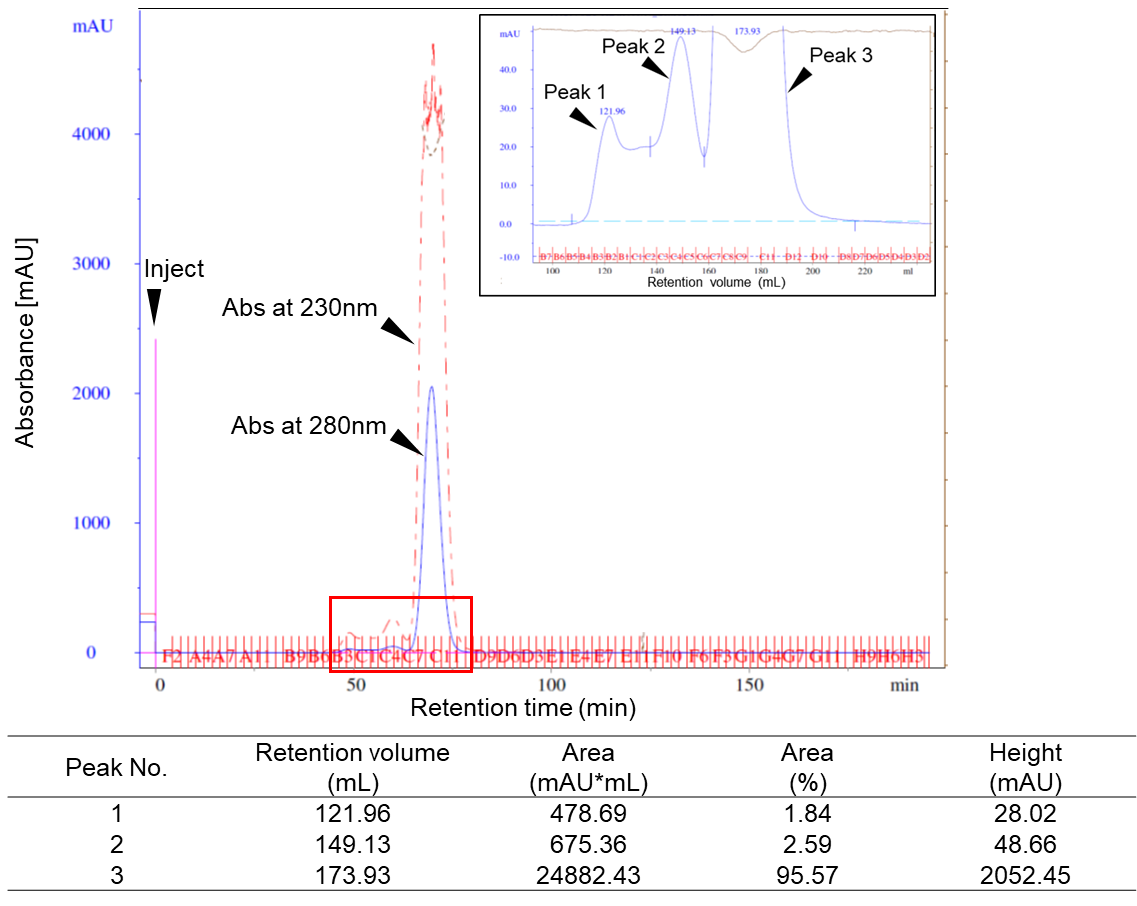
**

**Supplementary Figure S1 Fractionation of antibodies using size exclusion chromatography (SEC)|** Chromatogram and fractionation of antibodies using SEC. For antibody fractionation by SEC, antibody purified using affinity chromatography (118 mg) was injected into a HiLoad 26/60 Superdex 200 PG column equilibrated and eluted with PBS at a flow rate of 2.5 ml/min using AKTA explorer 100. Insert shows an enlarged view of the region of the SEC chromatogram enclosed by the red box. Each fraction was collected at 5 mL/fraction into a tube. Peak 1 (fraction number B4 to B1) in the chart was collected as the HMW fraction, peak 2 (fraction number C3 to C5) as the dimer fraction, and peak 3 (fraction number C7 to C12) as the monomer fraction.

**
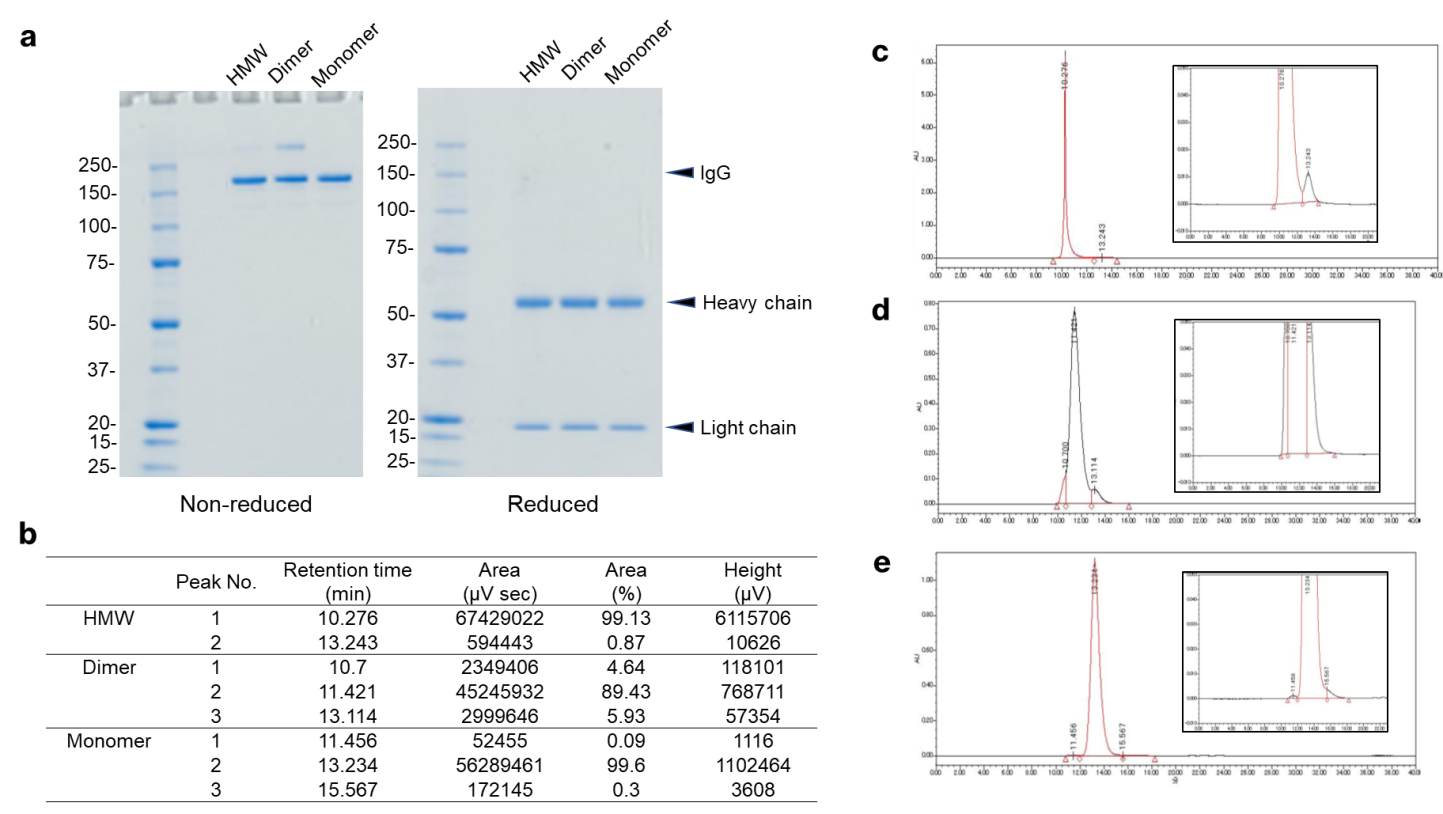
**

**Supplementary Figure S2 Purity analysis of monomer, dimer and high molecular weight (HMW) antibodies using SDS-PAGE and SEC|** a, SDS-PAGE analysis of monomer, dimer and HMW antibodies purified using SEC (Supplementary Fig.S1) under non-reduced (left) and reduced (right) conditions. Molecular weights (kDa) are indicated on the left of each gel. b, Table summarizing the results of SEC analysis using TSK gel G3000SW equilibrated and eluted with PBS at a flow rate of 0.5 mL/min. c–e, Chromatograms of HMW, dimer and monomer fractions. HMW shows a main peak at retention time 10.276 min with a purity of 99.13%. Dimers show a main peak at retention time 11.421 min with a purity of 89.43%. Monomers show a main peak at retention time 13.234 min with a purity of 99.6%.

**
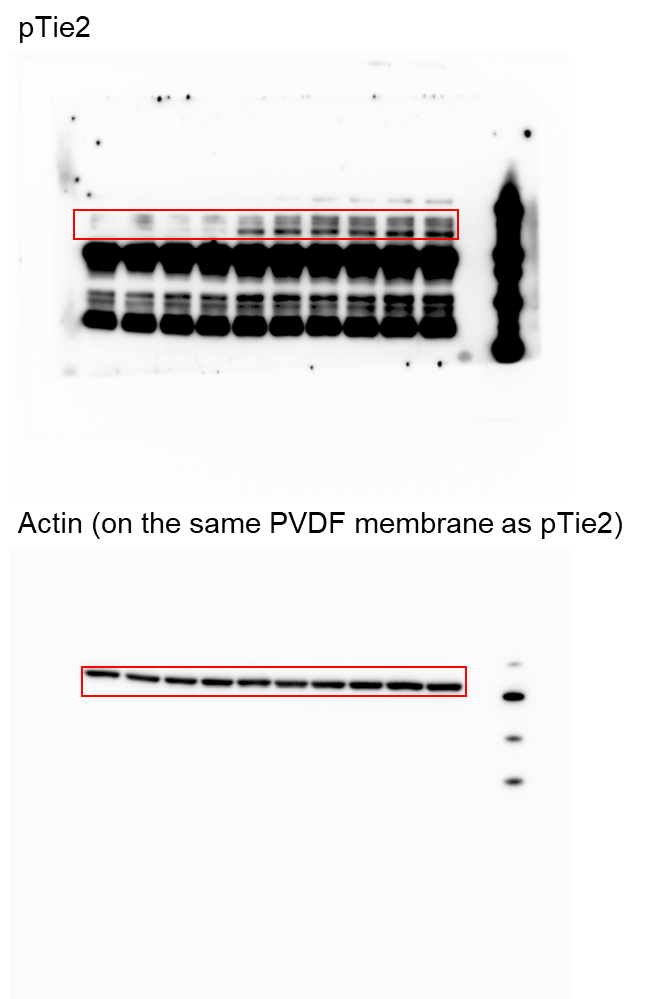
**

**Supplementary Figure S3 Full-size images of Western blots of phosphorylated Tie2 and actin shown in Fig. 2e.**


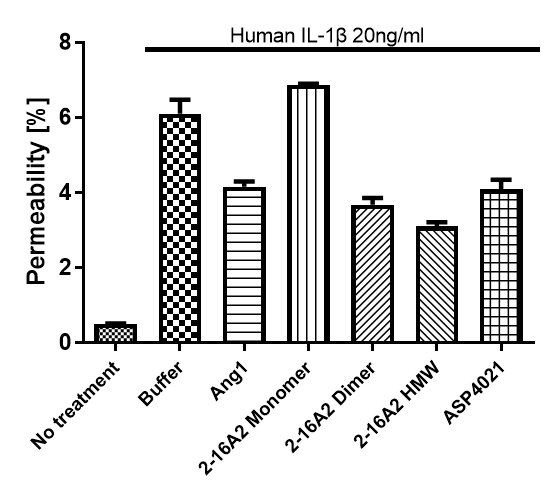


**Supplementary Figure S4 Inhibition of IL-1β-induced permeability by ASP4021 in HUVEC|** Permeability induced by human IL-1β in HUVEC was evaluated using the CultreCoat 96-well *in vitro* vascular permeability assay. Permeability (%) was calculated based on the relative fluorescence units; without cell/without FITC-dextran was defined as the background condition (0%) and without cell/with FITC-dextran as that with maximum permeability (100%). Data represent mean ± SEM.

| **Species** | **Immobilized amount (RU)** | **K_D_ (nmol L^-1^)** | **Geometric mean of K_D_ (nmol L^-1^)** | **95% Confidence interval of K_D_ (nmol L^-1^)** | ***k*_a1_ (mol^-1^ L s^-1^)** | **Geometric mean of *k*_a1_ (mol^-1^ L s^-1^)** | **95% Confidence interval of *k*_a1_ (mol^-1^ L s^-1^)** | ***k*_d1_ (s^-1^)** | **Geometric mean of *k*_d1_ (s^-1^)** | **95% Confidence interval of *k*_d1_ (s^-1^)** |
| --- | --- | --- | --- | --- | --- | --- | --- | --- | --- | --- |
| **Human** | 401 | 1.765 | 1.5 | 1.0–2.2 | 6.37E+05 | 6.00E+05 | 5.3E+5–6.9E+5 | 1.13E-03 | 8.90E-04 | 5.4E-4–1.5E-3 |
|  | 419 | 1.3 |  |  | 5.99E+05 |  |  | 7.79E-04 |  |  |
|  | 405.3 | 1.418 |  |  | 5.73E+05 |  |  | 8.12E-04 |  |  |
| **Mouse** | 438.9 | 0.825 | 1.0 | 0.65–1.6 | 6.93E+05 | 6.60E+05 | 4.9E+5–8.7E+5 | 5.72E-04 | 6.70E-04 | 4.4E-4–1.0E-3 |
|  | 406.7 | 1.149 |  |  | 5.77E+05 |  |  | 6.63E-04 |  |  |
|  | 393.5 | 1.124 |  |  | 7.12E+05 |  |  | 8.00E-04 |  |  |
| **Rat** | 413.8 | 1.404 | 1.3 | 0.81–2.2 | 5.76E+05 | 5.90E+05 | 5.2E+5–6.7E+5 | 8.09E-04 | 7.70E-04 | 5.4E-4–1.1E-3 |
|  | 399.4 | 1.549 |  |  | 5.63E+05 |  |  | 8.72E-04 |  |  |
|  | 409.6 | 1.062 |  |  | 6.21E+05 |  |  | 6.59E-04 |  |  |
| **Monkey** | 442.5 | 1.224 | 1.5 | 0.61–3.8 | 6.08E+05 | 5.90E+05 | 5.5E+5–6.3E+5 | 7.44E-04 | 9.00E-04 | 3.7E-4–2.1E-3 |
|  | 444 | 1.245 |  |  | 5.77E+05 |  |  | 7.19E-04 |  |  |
|  | 401.7 | 2.328 |  |  | 5.78E+05 |  |  | 1.35E-03 |  |  |

**Supplementary Table** | **Binding activity of ASP4021 to human, mouse, rat, and monkey Tie2**

Summary of binding kinetics of ASP4021 to human, mouse, rat, and monkey Tie2, analyzed using a Biacore system based on a surface plasmon resonance technique. The dissociation constant (K_D_), association rate constant (*k*_a1_) and dissociation rate constant (*k*_d1_) were calculated using Biacore T200 evaluation software version 3.0 (GE Healthcare Life Sciences) by fitting the results of sensorgrams obtained from a bivalent binding model (only Rmax was changed from “Fit global” to “Fit local”). For each antigen, the geometric mean and 95% confidence interval of K_D_, *k*_a1_, and *k*_d1_ were calculated using Microsoft® Excel 2010 SP2 (Microsoft).
